# Hospital-Physician Integration and Cardiac Rehabilitation Following Major Cardiovascular Events

**DOI:** 10.1001/jamanetworkopen.2024.62580

**Published:** 2025-03-03

**Authors:** Ngoc H. Thai, Brady Post, Gary J. Young, Md. Noor-E-Alam

**Affiliations:** 1 Center for Health Policy and Healthcare Research, Northeastern University, Boston, Massachusetts; 2 Department of Health Sciences, Bouve College of Health Sciences, Northeastern University, Boston, Massachusetts; 3 D’Amore McKim School of Business, Northeastern University, Boston, Massachusetts; 4 Department of Mechanical and Industrial Engineering, Northeastern University, Boston, Massachusetts

## Abstract

**Importance:**

Cardiac rehabilitation (CR) is a medically supervised program designed to improve heart health after a cardiac event. Despite its demonstrated clinical benefits, CR participation among eligible patients remains poor due to low referral rates and individual barriers to care.

**Objectives:**

To evaluate CR participation by patients who receive care from hospital-integrated physicians compared with independent physicians, and subsequently, to examine CR and recurrent cardiac hospitalizations.

**Design, Setting, and Participants:**

This retrospective cohort study evaluated Medicare Part A and Part B claims data from calendar years 2016 to 2019. All analyses were conducted between January 1 and April 30, 2024. Patients were included if they had a qualifying event for CR between 2017 and 2018, and qualifying events were identified using diagnosis codes on inpatient claims and procedure codes on outpatient and carrier claims. Eligible patients also had to continuously enroll in fee-for-service Medicare for 12 months or more before and after the index event. Physicians’ integration status and patients’ CR participation were determined during the 12-month follow-up period. The study covariates were ascertained during the 12 months before the index event.

**Exposure:**

Hospital-integration status of the treating physician during follow-up.

**Main Outcomes and Measures:**

Postindex CR participation was determined by qualifying procedure codes on outpatient and carrier claims.

**Results:**

The study consisted of 28 596 Medicare patients eligible for CR. Their mean (SD) age was 74.0 (9.6) years; 16 839 (58.9%) were male. A total of 9037 patients (31.6%) were treated by a hospital-integrated physician, of which 2995 (33.1%) received CR during follow-up. Logistic regression via propensity score weighting showed that having a hospital-integrated physician was associated with an 11% increase in the odds of receiving CR (odds ratio [OR], 1.11; 95% CI, 1.05-1.18). Additionally, CR participation was associated with a 14% decrease in the odds of recurrent cardiovascular-related hospitalizations (OR, 0.86; 95% CI, 0.81-0.91).

**Conclusions and Relevance:**

The findings of this cohort study suggest that hospital integration has the potential to facilitate greater CR participation and improve heart care. Several factors may help explain this positive association, including enhanced care coordination and value-based payment policies. Further research is needed to assess the association of integration with other appropriate high-quality care activities.

## Key Points

**Question:**

Is receiving care from hospital-integrated physicians after a cardiac event associated with a higher likelihood of receiving cardiac rehabilitation compared with independent physicians?

**Findings:**

In this cohort study of 28 596 Medicare beneficiaries with prior cardiac events, receiving care from a hospital-integrated physician was associated with an 11% increase in the odds of receiving cardiac rehabilitation. A secondary analysis found that cardiac rehabilitation was protective against recurrent hospitalizations.

**Meaning:**

The findings of this study suggest that hospital-physician integration has the potential to facilitate greater cardiac rehabilitation and improve outcomes for eligible patients.

## Teaser

This cohort study examines patient participation in cardiac rehabilitation programs when receiving care from physicians employed by hospitals compared with those in independent practice.

## Introduction

As hospitals have accelerated their acquisitions of physician groups over the past decade, medical care has become more structurally integrated.^1,2,3^ In theory, hospital employment of physicians, also referred to as hospital-physician integration, offers the potential to achieve greater efficiencies and enhance care quality while reducing costs.^4^ Proponents of such integration contend that a closer financial and organizational alignment between hospitals and physicians will lead to better clinical coordination for patient care.^5^ Furthermore, there is hope that hospital ownership of physician groups can reduce duplicative low-value care while also providing more high-value care through better sharing of information.^6^

Empirically, at least some research examining hospital-physician integration paints a different picture. Major studies have reported that hospital acquisitions of independent physician groups often lead to higher prices and spending.^1,7,8,9^ An underlying factor in this trend appears to be the transition to costly hospital-site care following acquisitions. There is evidence that hospital integration is associated with higher-intensity cardiac services^9^ and higher rates of inappropriate diagnostic imaging.^10^ This change in care practices following acquisitions can lead to low-value care and even adverse outcomes for patients.^9,10,11,12,13^

Still, there is also evidence that physician employment can offer hospitals a mechanism to perform well on potentially revenue-generating and quality-enhancing activities.^6,14,15,16,17^ Ho et al^14^ reported that breast cancer screening was more likely to occur in hospital-owned practices compared with independent practices. Similarly, Carlin et al^16^ observed that integration was associated with higher screening rates for breast, colorectal, and cervical cancers. These findings support the hypothesis that integrated systems are likely to prioritize care activities that clinically benefit patients while perhaps simultaneously improving the system’s bottom line.

In this study, we explored hospital-physician integration and outpatient cardiac rehabilitation (CR) and, subsequently, CR use and recurrent cardiovascular hospitalizations. Cardiac rehabilitation is a medically supervised program that offers structured exercise training, heart-healthy living education, and stress reduction counseling. Randomized clinical trials have documented the benefits of such programs in reducing the risk of mortality and secondary events and in improving patients’ health-related quality of life.^18,19,20,21,22,23^ Despite the evidence of its clinical benefits, overall rehabilitation participation rates remain markedly low, ranging from only 19% to 34% of eligible patients.^24^

One of the key reasons for the poor participation rates is the lack of physician referrals. Gurewich et al^25^ reported several factors likely responsible for the low referral rates, including capacity constraints, lack of automation in securing referrals, and the level of integration of CR within the hospital setting and physician community. Furthermore, CR programs generally involve complex care coordination among multiple specialists from inpatient and outpatient settings.^26^ Given the potential for alignment of resources and technology within vertically integrated systems, there is an opportunity here for hospital-employed physicians to outperform independent physicians in increasing CR participation. While several studies have reported a positive association between integrated systems and patients’ receipt of CR,^27,28^ to our knowledge, this study is among the first to specifically examine hospital-physician integration and access to CR. More broadly, our research may inform policymakers of potential areas of care in which integrated physicians might confer advantages over their independent counterparts.

## Methods

Because this cohort study used deidentified claims data from the Centers for Medicare & Medicaid Services, it was deemed exempt from review and the requirement for informed consent by Northeastern University’s Institutional Review Board. The study followed the Strengthening the Reporting of Observational Studies in Epidemiology (STROBE) reporting guideline.^29^

### Data Sources and Study Population

We analyzed Medicare Part A (inpatient) and Part B (outpatient and carrier) claims data from calendar year 2016 to 2019 using a retrospective cohort design. The study population consisted of patients who were considered eligible for CR following a major cardiovascular event during 2017 and 2018. In accordance with Medicare reimbursement policy, we included patients who had experienced one or more of the following events: an acute myocardial infarction (AMI), coronary artery bypass grafting, heart valve repair or replacement, percutaneous transluminal coronary angioplasty or coronary stenting, or a heart or lung transplant. Following Ritchey et al,^24^ we identified qualifying events based on the presence of specified *International Classification of Diseases and Related Health Problems, Tenth Revision, Clinical Modification* (*ICD-10-CM*) codes in the first and second positions or procedure codes in any location on inpatient claims or *Current Procedural Terminology* codes in any location on outpatient and carrier claims (eTable 1 in Supplement 1).

We defined the baseline period as the 12 months preceding the index event (ie, preindex) and the follow-up period as the 12 months following the index event (ie, postindex). Eligible patients had to also have continuous enrollment in fee-for-service Medicare and could not have been enrolled in privately administered Medicare health maintenance organizations (Medicare Advantage) in the 12 months both before and after the index event. The study design is illustrated in the Figure. All analyses were conducted between January 1 and April 30, 2024.

**Figure.**
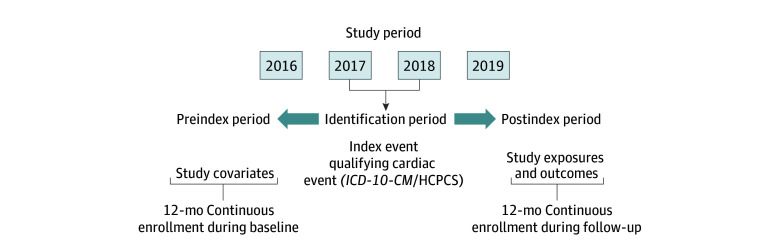
Study Design HCPCS indicates Healthcare Common Procedure Coding System; *ICD-10-CM*, *International Classification of Diseases and Related Health Problems, 10th Revision, Clinical Modification*.

### Study Outcomes

We ascertained the primary outcome, CR participation, based on the presence of any qualifying Current Procedural Terminology or Healthcare Common Procedure Coding System code on the outpatient and carrier claims during the follow-up period: 93798 (Healthcare Common Procedure Coding System with continuous electrocardiogram [ECG] monitoring), 93797 (CR without continuous ECG), G0422 (intensive CR with or without ECG monitoring with exercise), and G0423 (intensive CR with or without ECG monitoring without exercise).^30^

For the secondary outcome, recurrent cardiovascular hospitalizations, we identified inpatient claims that included specified cardiovascular *ICD-10-CM* codes as the principal diagnosis of the hospitalization. The list of *ICD-10-CM* codes for cardiovascular-related conditions is provided in eTable 2 in Supplement 1.

### Study Exposure

We identified the primary study exposure as whether a patient had a hospital-integrated primary care physician (PCP) or cardiologist in the 12 months following a cardiac event. We considered both PCPs and medical cardiologists due to their consequential roles in the management of heart disease. We attributed each patient to a physician using the patient attribution method developed by the Centers for Medicare & Medicaid Services.^31^ Additional details about this method are available in eMethods 1 in Supplement 1. We then determined the specialty code for that physician. Patients attributed to physicians who were not a PCP or cardiologist were excluded from the analysis. Approximately 10% of patients were removed due to this criterion.

We used the Medicare Data on Provider Practice and Specialty to determine hospital integration status. Guided by previous research,^32,33^ we identified physicians as hospital integrated if the legal name for their tax identification number referred to a hospital or a health system or 75% or more of their office and outpatient procedures were billed with hospital outpatient department place of service codes. An illustration of the study’s conceptual design describing the study outcomes and exposures is presented in eFigure 1 in Supplement 1.

### Study Covariates

Prior research has reported indicators such as comorbid conditions, race and ethnicity, ethnic minority status (eg, Asian, Hispanic, and non-Hispanic Black women), and high levels of service use are associated with both physician choice and CR participation.^34,35,36,37,38^ Race and ethnicity are included as demographic characteristics for a fuller picture of the study population. We developed an analytic dataset that contained more than 270 variables using Medicare Part A and B data. These variables included demographic characteristics (eg, age, sex, race and ethnicity), baseline health care costs and use measures (eg, inpatient, outpatient, office visits), baseline physician integration status, baseline Charlson Comorbidity Index status and its disease categories, and many other clinical indicators quantifying a patient’s burden of disease before the index event. We curated these clinical indicators using the Clinical Classification Software (CCS) program developed by the Healthcare Cost and Utilization Project (HCUP). The CCS program groups *ICD-10-CM* procedure and Healthcare Common Procedure Coding System codes into clinically meaningful categories, thereby providing the capacity to account for clinical conditions from a patient’s claims history.^39^

### Statistical Analysis

#### Descriptive Statistics

For our descriptive analysis of the study population, we compared patients who received treatment from hospital-integrated physicians and those who received treatment from independent physicians across several key variables representing patients’ demographic characteristics, baseline health care costs and service use, physician integration status, and comorbidities. We used χ^2^ tests to examine differences in proportions and the independent-sample, 2-tailed *t* test to test for differences in means between comparator groups. The R statistical software, version 4.4.0, was used for this study (R Foundation for Statistical Computing). The level of statistical significance was set at α .05.

#### Modeling Framework

We developed a 3-stage modeling process to evaluate hospital-physician integration and postindex CR participation, and subsequently, CR and recurrent cardiovascular hospitalizations. First, we used machine learning (ML) algorithms to identify potential confounders. Machine learning offers a useful approach to extract only the most informative factors from high-dimensional data.^40^ To this end, we developed 2 ML models—one for the exposure and one for the outcome—using several algorithms, including extreme gradient boosting, random forests, decision trees, and elastic net. We determined the best-performing algorithm using several model evaluation metrics, including accuracy, recall, and area under the curve (AUC). We subsequently identified the most important variables for both the exposure and the outcome.

In the second stage, we reduced the imbalances between the study groups through propensity score weighting using variables previously selected by ML. We then implemented a full-matching method to assign patients to subclasses based on the absolute differences in their propensity scores.^41^ Unlike k-to-1 matching, full matching does not exclude patients from the study population.

In the final stage, we conducted a logistic regression model for the propensity score–weighted sample to estimate the treatment effects of hospital-physician integration (primary exposure) and CR (secondary exposure). Details on the 3-stage modeling process are illustrated in eFigure 2 in Supplement 1.

#### Sensitivity Analysis

We performed a sensitivity analysis to estimate the outcome of integration of CR using an instrumental variable approach via a 2-stage generalized method of moments. The first stage was a logistic regression that estimated the integration status of the treating physician through an instrumental variable and the second stage estimated the exogenous outcome of integration with CR.^42^ Full details for the instrumental variable analysis are provided in eMethods 2 and eTable 3 in Supplement 1.

## Results

### Study Population and Descriptive Statistics

The study consisted of 28 596 patients who experienced a qualifying index event between 2017 and 2018, of whom 16 839 (58.9%) were male and 11 757 (41.1%) were female. The mean (SD) age was 74.0 (9.6) years. A total of 1.4% of the patients were Asian, 6.9% were Black, 1.3% were Hispanic, 0.6% were North American Native, 87.2% were White, 1.4% were of other race, and 1.2% were reported as unknown. Overall, 9037 patients (31.6%) received treatment from a hospital-integrated PCP (n = 4516) or medical cardiologist (n = 6749) during the 12-month follow-up period, and 19 559 (68.4%) received treatment from independent physicians. A total of 2228 patients received care from both. Of patients who received treatment from independent physicians, 5773 (29.5%) had at least one claim for CR services in the follow-up period. Among patients who received treatment from integrated physicians, 2995 (33.1%) had CR services during follow-up. Of 8769 patients who received CR, 78.3% initiated CR within 3 months after the index date.

We categorized health care costs and use incurred during preindex hospitalizations and visits as high (≥75th percentile), low (<75th percentile), and none. Patients who received treatment from integrated physicians had a greater share of high use of outpatient services in the preindex period compared with those who received treatment from independent physicians (40.3% vs 20.1%). Conversely, more patients of independent physicians incurred higher costs and use in the physician office setting than patients of integrated physicians (27.6% vs 19.4% for high office costs; 29.2% vs 19.6% for high office visits).

Patients who received treatment from integrated physicians and those of independent physicians appeared to have comparable levels of disease burden based on the Charlson Comorbidity Index (mean [SD], 1.2 [1.0]) in the integrated group compared with 1.1 (1.0) in the independent group. There was a slightly higher share of diabetes both with and without complications among patients who received treatment from integrated physicians (20.7% vs 19.9% for diabetes without complications, and 15.4% vs 10.9% for diabetes with complications). At baseline, patients who received treatment from integrated physicians also appeared to have higher shares of pulmonary disease (21.6% vs 16.7%), congestive heart failure (26.4% vs 19.9%), and AMI (13.4% vs 11.3%) than patients of independent physicians. Partial descriptive results are presented in Table 1. Full results are provided in eTable 4 in Supplement 1.

**Table 1.** Unadjusted Comparisons Between Patients of Hospital-Integrated vs Independent PCPs or Cardiologists

Characteristic	No. (%)		
	Patients of independent physicians post index (n = 19 559)	Patients of integrated physicians post index (n = 9037)	Overall (N = 28 596)
Age, mean (SD), y	74.2 (9.4)	73.5 (10.1)	74.0 (9.6)
Sex			
Male	11 528 (58.9)	5311 (58.8)	16 839 (58.9)
Female	8031 (41.1)	3726 (41.2)	11 757 (41.1)
Rural/urban classification			
Metropolitan/micropolitan	13613 (69.6)	5945 (65.8)	19 558 (68.4)
Rural	5923 (30.3)	3077 (34.0)	9000 (31.5)
Unknown	23 (0.1)	15 (0.2)	38 (0.1)
Race and ethnicity^a^			
Asian	301 (1.5)	100 (1.1)	401 (1.4)
Black	1287 (6.6)	692 (7.7)	1979 (6.9)
Hispanic	288 (1.5)	95 (1.1)	383 (1.3)
North American Native	77 (0.4)	93 (1.0)	170 (0.6)
White	17097 (87.4)	7838 (86.7)	24 935 (87.2)
Other^b^	283 (1.4)	115 (1.3)	398 (1.4)
Unknown	226 (1.2)	104 (1.2)	330 (1.2)
Region			
Midwest	3903 (20.0)	3008 (33.3)	6911 (24.2)
Northeast	3600 (18.4)	1690 (18.7)	5290 (18.5)
South	8607 (44.0)	3013 (33.3)	11 620 (40.6)
West	3400 (17.4)	1317 (14.6)	4717 (16.5)
Had a PCP (preindex)	15 408 (78.8)	6974 (77.2)	22 382 (78.3)
Had a cardiologist (preindex)	13 238 (67.7)	6138 (67.9)	19 376 (67.8)
Had an integrated PCP (preindex)			
No	14 920 (76.3)	3323 (36.8)	18243 (63.8)
Yes	488 (2.5)	3651 (40.4)	4139 (14.5)
No PCP	4151 (21.2)	2063 (22.8)	6214 (21.7)
Had an integrated cardiologist (preindex)			
No	12 339 (63.1)	2041 (22.6)	14380 (50.3)
Yes	899 (4.6)	4097 (45.3)	4996 (17.5)
No cardiologist	6321 (32.3)	2899 (32.1)	9220 (32.2)
CR use (postindex)	5773 (29.5)	2995 (33.1)	8768 (30.7)
CR use (preindex)	314 (1.6)	170 (1.9)	484 (1.7)
Time to initiation of CR, mo			
0-3	4508 (23.0)	2356 (26.1)	6864 (24.0)
3-6	947 (4.8)	471 (5.2)	1418 (5.0)
Charlson Comorbidity Index, mean (SD)	1.1 (1.0)	1.2 (1.0)	1.1 (1.0)

Abbreviations: CR, cardiac rehabilitation; PCP, primary care physician.

^a^ Race and ethnicity data were categorized directly from the data source.

^b^ As a direct pickup from claims data, no further breakdown is available for this category.

### Primary Objective: Vertical Integration and CR Participation

Following our 3-stage modeling process, the best ML algorithm for estimating postindex integration status was extreme gradient boosting as presented in eTable 5 in Supplement 1 (accuracy = 0.81; recall = 0.94; AUC = 0.85). The most important variables for this model included baseline physician integration status and whether a patient had a cardiologist or PCP before the index event. Baseline integration status of either the PCP or cardiologist was the highest ranking factor in estimating postindex integration status based on the gain metric (Table 2).

**Table 2.** Most Important Variables for Postindex Physician Integration Status

Ranking	Variable	Gain	Frequency, %
1	Had an integrated cardiologist (preindex)	0.426	6.803
2	Had an integrated PCP (preindex)	0.424	6.803
3	Had a cardiologist (preindex)	0.054	4.082
4	Had a PCP (preindex)	0.026	1.361
5	No CVD office visit (preindex)	0.011	2.041
6	Region, south	0.006	2.721
7	Had no PCP (preindex)	0.004	1.361
8	Low use of OP visits (preindex)	0.003	2.041
9	No CVD OP visit charges (preindex)	0.003	0.680
10	No OP visit charges (preindex)	0.002	1.361
11	Age	0.002	3.401
12	Region, west	0.002	2.041
13	No office visits (preindex)	0.002	0.680
14	Low use of CVD OP visits (preindex)	0.002	2.041
15	Rural residence	0.002	2.041
16	Prophylactic vaccinations and inoculations	0.001	2.041
17	Low OP visit charges (preindex)	0.001	1.361
18	Electrographic cardiac monitoring	0.001	0.680
19	No OP visit charges (preindex)	0.001	1.361
20	Low CVD office visit charges (preindex)	0.001	1.361

Abbreviations: CVD, cardiovascular disease; OP, outpatient; PCP, primary care physician.

For the ML model estimating CR participation, the best-performing algorithm (extreme gradient boosting) had an accuracy of 0.64, recall of 0.90, and AUC of 0.70 (eTable 6 in Supplement 1). The top 5 variables for this model were having coronary artery bypass grafting as the index cardiac event, age, having an AMI as the index event, use of hospital transportation for emergency or nonemergency care, and having a percutaneous transluminal coronary angioplasty as the index event. The top 20 variables of both models are presented in Table 2 and Table 3.

**Table 3.** Most Important Variables for Postindex CR Use

Ranking	Variable	Gain	Frequency, %
1	CABG (index event)	0.132	1.840
2	Age	0.124	7.055
3	AMI (index event)	0.110	2.301
4	Use of transportation for emergency and nonemergency care (preindex)	0.058	1.227
5	PTCA (index event)	0.030	1.687
6	Diagnostic cardiac catheterization, coronary arteriography (preindex)	0.030	1.227
7	Excision of skin lesion (preindex)	0.026	1.380
8	Region, south	0.023	2.301
9	Physical therapy exercises, manipulation, and other procedures (preindex)	0.023	1.380
10	Region, northeast	0.021	2.147
11	Sex, female	0.020	1.534
12	Region, west	0.019	1.380
13	CR (preindex)	0.019	1.534
14	No inpatient charges (preindex)	0.018	0.460
15	Ophthalmologic and otologic diagnosis and treatment (preindex)	0.018	1.380
16	Heart valve repair and replacement (preindex)	0.017	1.534
17	Peritoneal dialysis (preindex)	0.011	0.460
18	Other nonsurgical therapeutic procedures on musculoskeletal system (preindex)	0.011	0.767
19	PTCA (preindex)	0.010	0.307
20	Rural residence	0.010	1.380

Abbreviations: AMI, acute myocardial infarction; CABG, coronary artery bypass graft; CR, cardiac rehabilitation; PTCA, percutaneous transluminal coronary angioplasty.

Subsequently, we constructed propensity score weights using the most important variables identified by ML. Propensity score matching reduced the imbalances between the study groups to a great extent (standardized mean difference = 0, variance ratio = 1). After matching, we conducted logistic regression modeling on the propensity score–weighted sample to estimate the possible association between integration and CR use. The regression results indicated that having a hospital-integrated treating physician (PCP or cardiologist) was associated with an 11% increase in the odds of receiving CR services among eligible patients (odds ratio [OR], 1.11; 95% CI, 1.05-1.18). Results from our sensitivity analysis using an instrumental variable via 2-stage generalized substitution estimators in eMethods 2 in Supplement 1 bolstered this finding, suggesting an association between integration and CR (OR, 1.26; 95% CI, 1.05-1.52). Our model also showed an association between sex and CR participation. Specifically, female patients had 24% lower odds of receiving CR compared with male patients (OR, 0.76; 95% CI, 0.72-0.80). The full logistic regression results are provided in Table 4.

**Table 4.** Logistic Regression Results for Postindex CR Use

Variable	OR (95% CI)
Integrated PCP or cardiologist (postindex)	1.11 (1.05-1.18)
Female sex	0.76 (0.72-0.80)
Region, northeast	0.49 (0.46-0.53)
Region, south	0.51 (0.48-0.55)
Region, west	0.55 (0.50-0.60)
Age	0.98 (0.98-0.99)
RUCA, rural	0.83 (0.78-0.88)
CABG at index	3.86 (3.48-4.29)
AMI at index	1.11 (1.01-1.23)
Use of transportation for emergency/nonemergency care	0.56 (0.53-0.60)
PTCA index event	1.61 (1.48-1.75)
Diagnostic cardiac catheterization, coronary arteriography	1.51 (1.38-1.66)
Excision of skin lesion	1.43 (1.35-1.52)
Physical therapy exercises, manipulation, and procedures	1.49 (1.39-1.59)
Preindex CR use	2.64 (2.18-3.20)
Low inpatient charges (preindex)	1.03 (0.94-1.14)
No inpatient charges (preindex)	1.48 (1.37-1.60)
Ophthalmologic and otologic diagnosis and treatment	1.39 (1.31-1.47)
Heart valve replacement and repair index event	2.06 (1.86-2.29)
Peritoneal dialysis	0.29 (0.24-0.36)
Other nonsurgical therapeutic procedures on musculoskeletal system	1.45 (1.32-1.60)
Preindex PTCA procedure	0.72 (0.64-0.81)

Abbreviations: AMI, acute myocardial infarction; CABG, coronary artery bypass grafting; CR, cardiac rehabilitation; OR, odds ratio; PCP, primary care physician; PTCA, percutaneous transluminal coronary angioplasty; RUCA, rural-urban commuting area.

### Secondary Objective: CR Participation and Recurrent Cardiovascular Hospitalizations

For the secondary objective, we similarly implemented ML models to identify important variables in the examination of CR and recurrent hospitalizations. The performance metrics presented in eTable 7 in Supplement 1 indicate that elastic net was the best-performing algorithm for estimating recurrent hospitalizations (accuracy = 0.55; recall = 0.96; AUC = 0.65). The most important variables for both CR and recurrent hospitalizations included prior use of transportation for emergency or nonemergency care, prior occurrence of diagnostic cardiac catheterization, having a heart valve repair or replacement as the index event, having an AMI as the index event, and baseline inpatient hospitalization costs. The top 20 variables for this model are reported in eTable 8 in Supplement 1.

After propensity score matching, the 2 comparator groups appeared to achieve good balances across all identified confounding variables (standardized mean difference = 0; variance ratio = 1). Our final logistic regression model in eTable 9 in Supplement 1 shows that participation in a CR program after an index cardiac event was associated with 14% lower odds of having a recurrent cardiovascular-related hospitalization (OR, 0.86; 95% CI, 0.81-0.91).

## Discussion

Cardiac rehabilitation is useful for heart disease care: it reduces mortality and rehospitalizations and improves physical function and health-related quality of life.^19,20,21,22^ Despite these clinical benefits, participation among eligible patients is pervasively low. However, physicians play a key role in educating and referring patients. Prior work has reported that hospital-integrated physicians can steer patients toward preferred sites and services.^9,10,11^ Our results suggest they might also steer patients toward CR more effectively than independent physicians.

Both theory and empirical evidence help to explain why this may be the case. First, CR is a complex program that typically involves a team of multidisciplinary specialists.^43^ Integrated health systems might be better staffed than independent practices for coordinating care for patients. Furthermore, with centralized technology and information sharing, integrated systems may better facilitate care transitions from inpatient to outpatient settings. Grace et al^44^ reported that CR referral rates could be almost tripled by using an automatic referral system. As hospitals continue to expand their digital capabilities, hospital-integrated physicians may outperform independent physicians in activities that rely on technological investment. Second, CR services generate more revenue for hospitals than for independent physicians. As of 2020, the reimbursement for a traditional CR session in an independent office was $70 vs a hospital outpatient department for which it was $116.^45^ This may encourage hospital-based physicians to refer patients to CR. Third, value-based payment models from Medicare and commercial payers incentivize hospitals to reduce adverse outcomes in cardiovascular care. For example, the Hospital Readmissions Reduction Program targets conditions, including AMI, heart failure, and coronary artery bypass grafting, and the Hospital Value-Based Purchasing Program incorporated mortality rates from AMI and heart failure into its hospital performance measures. For these reasons, hospitals may encourage their physicians to steer patients toward CR. Our finding that CR was associated with fewer subsequent hospitalizations adds to the evidence that such a strategy can work.

Consolidation of hospitals and physicians has become a fixture of the US health care system. While a growing body of research has found evidence of use of low-quality, inappropriate care resulting from this trend, our study results suggest that for certain activities, patient steering may also lead to higher use of appropriate high-value care. Cardiac rehabilitation falls into the care category of quality-improvement and potential profitability for hospitals. Whether other services that belong in this quadrant would also see improvement from vertical integration is worthy of closer scrutiny.

### Limitations

Our study is not without limitations. Despite our attempt to account for potential confounders by including a large number of variables from the claims data and using ML to select appropriate variables, some residual confounding and selection bias likely persisted. As is often the case with observational studies that use administrative claims databases, we lack information on patients’ motivations to enroll in and adhere to CR programs. Similarly, a patient’s choice of physician likely depends in part on factors not available in the claims data. However, our instrumental variable analysis presents important findings with regard to integration and CR use.

## Conclusions

In this cohort study of patients who were eligible for CR following a major cardiac event, we explored hospital-physician integration and cardiovascular care quality. Eligible patients who received outpatient care from hospital-integrated PCPs or cardiologists were more likely to participate in CR programs than those receiving care from independent physicians. Our study further suggests that participation in CR programs may be protective against secondary cardiovascular-related hospitalizations, underscoring the positive association between CR and patient outcomes. As antitrust enforcers increase regulatory scrutiny of vertical integration activities, further research is needed to broaden the evidence on consolidation in care quality.

## Notes

Supplement 1. **eTable 1.** Sources Used for Qualifying Cardiac Events **eTable 2.** Claims-Based Definitions of Common Cardiovascular Conditions to Identify Cardiovascular-Related Service Use, Costs, and Hospitalizations **eMethods 1.** Physician Attribution Methodology Developed by the Centers for Medicare and Medicaid Services **eFigure 1.** Illustrative Diagram of the Study’s Primary and Secondary Objectives **eFigure 2.** Illustration of the 3-Stage Modeling Process for Treatment Effect Estimation **eMethods 2.** Using Instrumental Variable Analysis to Estimate the Effect of Integration on Cardiac Rehab Participation **eTable 3.** Odds Ratios and 95% Confidence Intervals Estimated by the Two-Stage Generalized Predictor Substitution Approach for the Primary Study Exposure (ie, Receiving Care From an Integrated Physician) and Other Study Covariates **eTable 4.** Full Results of Unadjusted Comparisons Between Patients of Hospital-Integrated Primary Care Physicians or Cardiologists and Patients of Independent Primary Care Physicians or Cardiologists **eTable 5.** Model Accuracy Metrics From Machine Learning Algorithms Predicting the Primary Exposure (ie, Receiving Care From an Integrated Primary Care Physician or Cardiologist) **eTable 6.** Model Accuracy Metrics From Machine Learning Algorithms Predicting the Primary Outcome (ie, Receiving Post-Index Cardiac Rehab Services) **eTable 7.** Model Accuracy Metrics From Machine Learning Models Predicting the Secondary Exposure (ie, the Likelihood of Cardiac Rehab Participation) **eTable 8.** Most Important Variables Identified by the Elastic Net Algorithm for Predicting Recurrent Cardiovascular-Related Hospitalizations (ie, the Secondary Outcome) **eTable 9.** Odds Ratios and 95% Confidence Intervals From Logistic Regression Model for Recurrent Cardiovascular-Related Hospitalizations (ie, the Secondary Outcome)
eReferences

Supplement 2.
Data Sharing Statement
