# Missing Author Initial in Byline

**DOI:** 10.1001/jamanetworkopen.2025.8695

**Published:** 2025-03-28

In the Original Investigation titled “Hospital-Physician Integration and Cardiac Rehabilitation Following Major Cardiovascular Events,”^1^ published March 3, 2025, an author initial was missing from the byline. The third author listed as “Gary Young, JD, PhD” should have been “Gary J. Young, JD, PhD.” This article has been corrected.^1^
