# Supplementary material for: Hospital-Physician Integration and Cardiac Rehabilitation Following Major Cardiovascular Events
Source: JAMA Netw Open. 2025 Mar 3;8(3):e2462580. doi: 10.1001/jamanetworkopen.2024.62580 (PMC11877175; doi:10.1001/jamanetworkopen.2024.62580)
Supplement: Supplement 1. — eTable 1. Sources Used for Qualifying Cardiac Events eTable 2. Claims-Based Definitions of Common Cardiovascular Conditions to Identify Cardiovascular-Related Service Use, Costs, and Hospitalizations eMethods 1. Physician Attribution Methodology Developed by the Centers for Medicare and Medicaid Services eFigure 1. Illustrative Diagram of the Study’s Primary and Secondary Objectives eFigure 2. Illustration of the 3-Stage Modeling Process for Treatment Effect Estimation eMethods 2. Using Instrumental Variable Analysis to Estimate the Effect of Integration on Cardiac Rehab Participation eTable 3. Odds Ratios and 95% Confidence Intervals Estimated by the Two-Stage Generalized Predictor Substitution Approach for the Primary Study Exposure (ie, Receiving Care From an Integrated Physician) and Other Study Covariates eTable 4. Full Results of Unadjusted Comparisons Between Patients of Hospital-Integrated Primary Care Physicians or Cardiologists and Patients of Independent Primary Care Physicians or Cardiologists eTable 5. Model Accuracy Metrics From Machine Learning Algorithms Predicting the Primary Exposure (ie, Receiving Care From an Integrated Primary Care Physician or Cardiologist) eTable 6. Model Accuracy Metrics From Machine Learning Algorithms Predicting the Primary Outcome (ie, Receiving Post-Index Cardiac Rehab Services) eTable 7. Model Accuracy Metrics From Machine Learning Models Predicting the Secondary Exposure (ie, the Likelihood of Cardiac Rehab Participation) eTable 8. Most Important Variables Identified by the Elastic Net Algorithm for Predicting Recurrent Cardiovascular-Related Hospitalizations (ie, the Secondary Outcome) eTable 9. Odds Ratios and 95% Confidence Intervals From Logistic Regression Model for Recurrent Cardiovascular-Related Hospitalizations (ie, the Secondary Outcome) eReferences [file jamanetwopen-e2462580-s001.pdf]

Supplementary Online Content

Thai N, Post B, Young GJ, Noor-E-Alam Md. Hospital-physician integration and cardiac rehabilitation following major cardiovascular events. *JAMA Netw Open*. 2025;8(3):e2462580. doi:10.1001/jamanetworkopen.2024.62580

eTable 1. Sources Used for Qualifying Cardiac Events

eTable 2. Claims-Based Definitions of Common Cardiovascular Conditions to Identify Cardiovascular-Related Service Use, Costs, and Hospitalizations

eMethods 1. Physician Attribution Methodology Developed by the Centers for Medicare and Medicaid Services

eFigure 1. Illustrative Diagram of the Study's Primary and Secondary Objectives

eFigure 2. Illustration of the 3-Stage Modeling Process for Treatment Effect Estimation

eMethods 2. Using Instrumental Variable Analysis to Estimate the Effect of Integration on Cardiac Rehab Participation

eTable 3. Odds Ratios and 95% Confidence Intervals Estimated by the Two-Stage Generalized Predictor Substitution Approach for the Primary Study Exposure (ie, Receiving Care From an Integrated Physician) and Other Study Covariates

eTable 4. Full Results of Unadjusted Comparisons Between Patients of Hospital-Integrated Primary Care Physicians or Cardiologists and Patients of Independent Primary Care Physicians or Cardiologists

eTable 5. Model Accuracy Metrics From Machine Learning Algorithms Predicting the Primary Exposure (ie, Receiving Care From an Integrated Primary Care Physician or Cardiologist)

eTable 6. Model Accuracy Metrics From Machine Learning Algorithms Predicting the Primary Outcome (ie, Receiving Post-Index Cardiac Rehab Services)

eTable 7. Model Accuracy Metrics From Machine Learning Models Predicting the Secondary Exposure (ie, the Likelihood of Cardiac Rehab Participation)

eTable 8. Most Important Variables Identified by the Elastic Net Algorithm for Predicting Recurrent Cardiovascular-Related Hospitalizations (ie, the Secondary Outcome)

eTable 9. Odds Ratios and 95% Confidence Intervals From Logistic Regression Model for Recurrent Cardiovascular-Related Hospitalizations (ie, the Secondary Outcome)

eReferences

This supplementary material has been provided by the authors to give readers additional information about their work.

© 2025 Thai N et al. *JAMA Network Open.*

eTable 1. Sources Used for Qualifying Cardiac Events
Healthcare Common Procedure Coding System (HCPCS), International Classification of Disease, Tenth Revision, Procedure Codes (ICD-10 PCS), and International Classification of Disease, Tenth Revision, Clinical Modification (ICD-10 CM)

Cardiac Event	Code Type	Code
**Acute Myocardial Infarction (AMI)**	ICD-10 CM	I210, I2101, I2102, I2109, I211, I2111, I2119, I212, I2121, I2129, I213, I214, I219, I21A1, I21A9, I220, I221, I222, I228, I229
**Coronary Artery Bypass Graft (CABG)**	ICD-10 PCS	0210X, 0211X, 0212X, 0213X
	HCPCS	33510, 33511, 33512, 33513, 33514, 33516, 33517, 33518, 33519, 33521, 33522, 33523, 33530, 33533, 33534, 33535, 33536, 33572, 35600, S2205, S2206, S2207, S2208, S2209
**Heart Lung Transplant**	ICD-10 PCS	02YA0Z0, 02YA0Z1, 02YA0Z2, 0BYM0Z0, 0BYM0Z1, 0BYM0Z2, 02YA0Z0, 02YA0Z1, 02YA0Z2, 02RK0JZ, 02RL0JZ, 02WA0JZ, 02WA0JZ
	HCPCS	33945, 33927, 33928, 0051T, 0052T, 0053T
**Percutaneous Transluminal Coronary Angioplasty (PTCA)**	ICD-10 PCS	02703ZZ, 02704ZZ, 02713ZZ, 02714ZZ, 02723ZZ, 02724ZZ, 02733ZZ, 02734ZZ, 3E07017, 3E070PZ, 3E07317, 3E073PZ, 02700ZZ, 02710ZZ, 02720ZZ, 02730ZZ, 02C00ZZ, 02C10ZZ, 02C20ZZ, 02C30ZZ, 02C03ZZ, 02C04ZZ, 02C13ZZ, 02C14ZZ, 02C23ZZ, 02C24ZZ, 02C33ZZ, 02C34ZZ
	HCPCS	92920, 92921, 92924, 92925, 92929, 92933, 92934, 92937, 92938, 92941, 92943, 92944, 92973, 92974
Heart Valve Repair/Replacement	ICD-10 PCS	027F, 027G, 027H, 027J, 02CF, 02CG, 02CH, 02CJ, 02NF, 02NG, 02NH, 02NJ, 02QF, 02QG, 02QH, 02QJ, 02RF, 02RG, 02RH, 02RJ, 02TH, 02VG, 02UF, 02UG, 02UH, 02UJ
	HCPCS	33361, 33362, 33363, 33364, 33365, 33366, 33367, 33368, 33369, 33370, 33371, 33372, 33373, 33374, 33375, 33376, 33377, 33378, 33379, 33380, 33381, 33382, 33383, 33384, 33385, 33386, 33387, 33388, 33389, 33390, 33391, 33392, 33393, 33394, 33395, 33396, 33397, 33398, 33399, 33400, 33401, 33402, 33403, 33404, 33405, 33406, 33407, 33408, 33409, 33410, 33411, 33412, 33413, 33414, 33415, 33416, 33417, 33418, 33419, 33420, 33421, 33422, 33423, 33424, 33425, 33426, 33427, 33428, 33429, 33430, 33460, 33461, 33462, 33463, 33464, 33465, 33466, 33467, 33468, 33470, 33471, 33472, 33473, 33474, 33475, 33476, 33477, 33478, 33361, 33362, 33363, 33364, 33365, 33366, 33367, 33368, 33369, 33370, 33371, 33372, 33373, 33374, 33375, 33376, 33377, 33378, 33379, 33380, 33381, 33382, 33383, 33384, 33385, 33386, 33387, 33388, 33389, 33390, 33391, 33392, 33393, 33394, 33395, 33396, 33397, 33398, 33399, 33400, 33401, 33402, 33403, 33404, 33405, 33406, 33407, 33408, 33409, 33410, 33411, 33412, 33413, 33414, 33415, 33416, 33417, 33418, 33419, 33420, 33421, 33422, 33423, 33424, 33425, 33426, 33427, 33428, 33429, 33430, 33460, 33461, 33462, 33463, 33464, 33465, 33466, 33467, 33468, 33470, 33471, 33472, 33473, 33474, 33475, 33476, 33477, 33478

© 2025 Thai N et al. *JAMA Network Open.*

eTable 2. Claims-based Definitions of Common Cardiovascular Conditions to Identify Cardiovascular-related Service Use, Costs, and Hospitalizations

Diagnosis	Type	ICD-10 Code
Heart attack	STEMI	I21.01, I21.02, I21.09, I21.11, I21.19, I21.21, I21.29, I21.3
	NSTEMI	I21.4
	Unspecified	I21.9
	Type 2	I21.A1
	Other Types	I21.A9
Stroke	TIA	G45.0, G45.1, G45.2, G45.8, G45.9, I67.81, I67.82, I67.841, I67.848, I67.89
	Ischemic & hemorrhagic stroke	60.00, I60.01, I60.02, I60.10, I60.11, I60.12, I60.2, I60.20, I60.21, I60.22, I60.30, I60.31, I60.32, I60.4, I60.50, I60.51, I60.52, I60.6, I60.7, I60.8, I60.9, I61.0, I61.1, I61.2, I61.3, I61.4, I61.5, I61.6, I61.8, I61.9, I63.00, I63.011, I63.012, I63.013, I63.019, I63.02, I63.031, I63.032, I63.033, I63.039, I63.09, I63.10, I63.111, I63.112, I63.113, I63.119, I63.12, I63.131, I63.132, I63.133, I63.139, I63.19, I63.20, I63.211, I63.212, I63.213, I63.219, I63.22, I63.231, I63.232, I63.233, I63.239, I63.29, I63.30, I63.311, I63.312, I63.313, I63.319, I63.321, I63.322, I63.323, I63.329, I63.331, I63.332, I63.333, I63.339, I63.341, I63.342, I63.343, I63.349, I63.39, I63.40, I63.411, I63.412, I63.413, I63.419, I63.421, I63.422, I63.423, I63.429, I63.431, I63.432, I63.433, I63.439, I63.441, I63.442, I63.443, I63.449, I63.49, I63.50, I63.511, I63.512, I63.513, I63.519, I63.521, I63.522, I63.523, I63.529, I63.531, I63.532, I63.533, I63.539, I63.541, I63.542, I63.543, I63.549, I63.59, I63.6, I63.81, I63.89, I63.9
	Other stroke symptoms	G46.0, G46.1, G46.2, G46.3, G46.4, G46.5, G46.6, G46.7, G46.8, G97.31, G97.32, I66.01, I66.02, I66.03, I66.09, I66.11, I66.12, I66.13, I66.19, I66.21, I66.22, I66.23, I66.29, I66.3, I66.8, I66.9, I97.810, I97.811, I97.820, I97.821
Other Conditions	Other most frequently found cardiovascular disease codes	A5201, B3322, C380, D151, G454, G9340, G9341, G9349, G9389, G939, G968, G969, G980, G988, I011, I018, I019, I050, I051, I052, I058, I059, I060, I061, I062, I068, I069, I071, I078, I079, I080, I081, I082, I083, I088, I089, I0981, I0989, I099, I10, I110, I119, I130, I1310, I132, I150, I151, I152, I158, I159, I160, I161, I169, I200, I201, I208, I209, I236, I240, I241, I248, I249, I2510, I25110, I25111, I25118, I25119, I252, I253, I2541, I255, I256, I25700, I25701, I25708, I25709, I25710, I25718, I25719, I25720, I25721, I25728, I25729, I25739, I25750, I25758, I25759, I25790, I25791, I25798, I25799, I25810, I25811, I25812, I2582, I2583, I2584, I2589, I259, I270, I271, I2720, I2721, I2781, I2789, I279, I281, I288, I289, I300, I301, I308, I309, I311, I312, I313, I314, I318, I319, I32, I330, I339, I340, I341, I342, I348, I349, I350, I351, I352, I358, I359, I360, I361, I362, I368, I369, I370, I371, I372, I379, I38, I39, I400, I401, I41, I420, I421, I422, I423, I425, I426, I427, I428, I429, I43, I440, I441, I442, I4430, I4439, I444, I447, I450, I4510, I4519, I452, I453, I454, I455, I456, I4581, I4589, I459, I462, I468, I469, I470, I471, I472, I479, I480, I481, I482, I483, I484, I4891, I4892, I4901, I4902, I491, I492, I493, I4940, I4901, I4902, I491, I493, I4949, I495, I498, I499, I501, I5020, I5021, I5022, I5023, I5030, I5031, I5032, I5033, I5040, I5041, I5042, I5043, I50810, I509, I510, I511, I513, I514, I515, I517, I5181, I5189, I519, I52, I6200, I6201, I6202, I6203, I621, I629, I6501, I6502, I6503, I6509, I651, I6521, I6522, I6523, I6529, I658, I659, I672, I6781, I6782, I6783, I679, I680, I700.

Abbreviations. ICD-10, International Classification of Disease, Tenth Revision; NSTEMI, non-ST-elevation myocardial infarction; STEMI, ST-elevation myocardial infarction; TIA, transient ischemic attack.

© 2025 Thai N et al. *JAMA Network Open.*

eMethods 1. Physician Attribution Methodology Developed by the Centers for Medicare and Medicaid Services (CMS)

We attributed each patient to a physician (either a primary care physician or a cardiologist) using the patient attribution methodology developed by CMS. This methodology accounted for the share of allowed charges for primary care services during a patient's 12-month follow-up period. In alignment with CMS' methodology, we identified primary care services using Healthcare Common Procedure Coding System (HCPCS) codes in any location on the carrier (professional) claims. We identified the physician with the largest share of allowed charges for such services during the follow-up period as a patient's treating physician.

Healthcare Common Procedure Coding Systems (HCPCS) primary care service codes

HCPCS Code	Description
99201–99205	New patient, office, or other outpatient visit
99211–99215	Established patient, office, or other outpatient visit
99304–99306	New patient, nursing facility care
99307–99310	Established patient, nursing facility care
99315–99316	Established patient, discharge day management service
99318	Established patient, other nursing facility service
99324–99328	New patient, domiciliary or rest home visit
99334–99337	Established patient, domiciliary or rest home visit
99339–99340	Established patient, physician supervision of patient (patient not present) in home, domiciliary, or rest home
99347–99350	New patient, home visit
99347–99350	Established patient, home visit
G0402	Initial Medicare visit
G0438	Annual wellness visit, initial
G0439	Annual wellness visit, subsequent
G0463	Hospital outpatient clinic visit (Electing Teaching Amendment hospitals only)

© 2025 Thai N et al. *JAMA Network Open.*

eFigure 1. Illustrative Diagram of the Study's Primary and Secondary Objectives

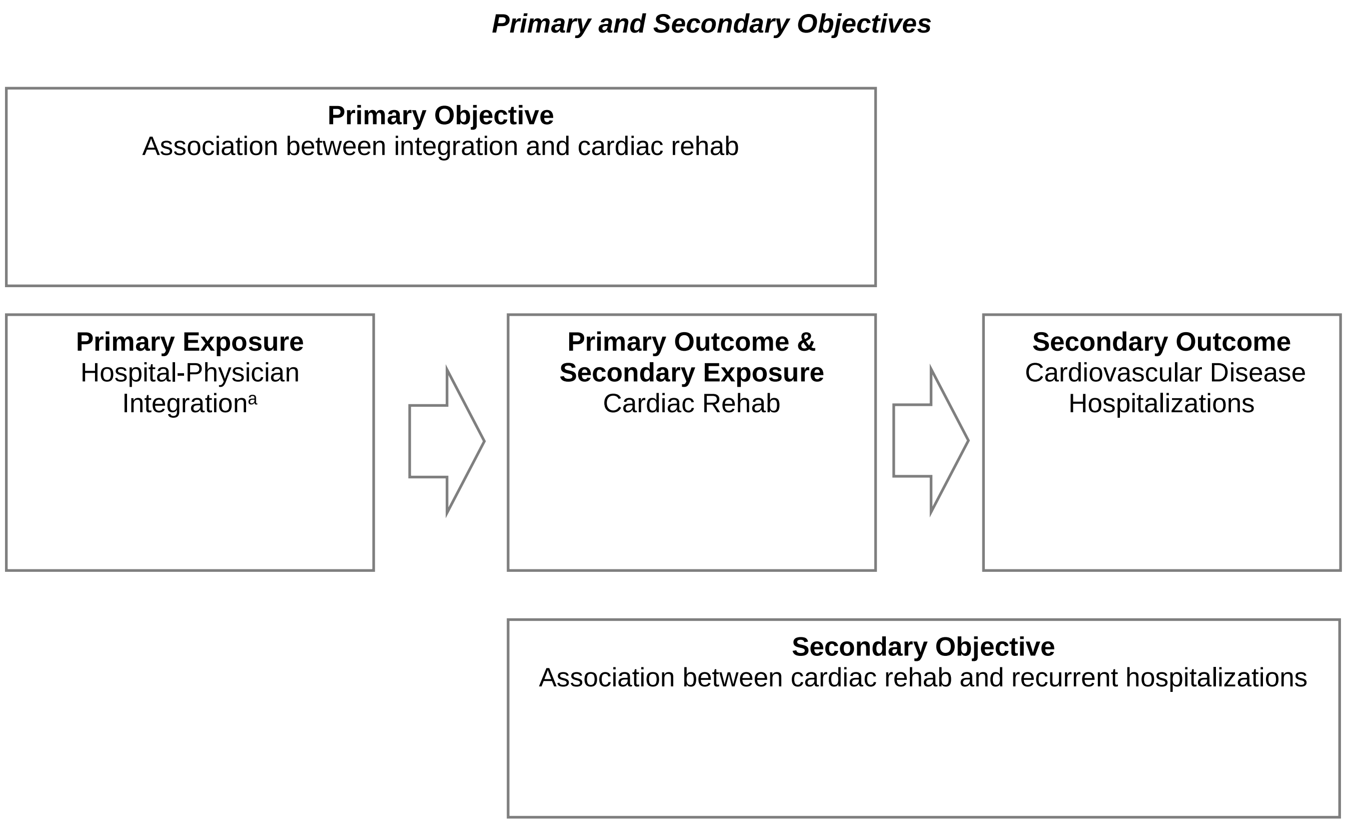

[a]Guided by prior research, we identified physicians as hospital-integrated if (1) the legal name for their tax identification number (TIN) referred to a hospital or a health system, or (2) 75 percent or more of their office and outpatient procedures were billed with hospital outpatient department place of service codes using the Medicare Data on Provider Practice and Specialty. Using the TIN approach, Ho et al. (2020) determined that about 88 percent of the physicians commonly identified in MD-PPAS and a commercial claims database had the same ownership type.[1] The claims-based approach developed by Neprash and colleagues, which identified integration status based on a physician's share of hospital outpatient claims, supplemented the remainder of physicians.[2]

© 2025 Thai N et al. *JAMA Network Open.*

eFigure 2. Illustration of the 3-stage modeling process for treatment effect estimation

s

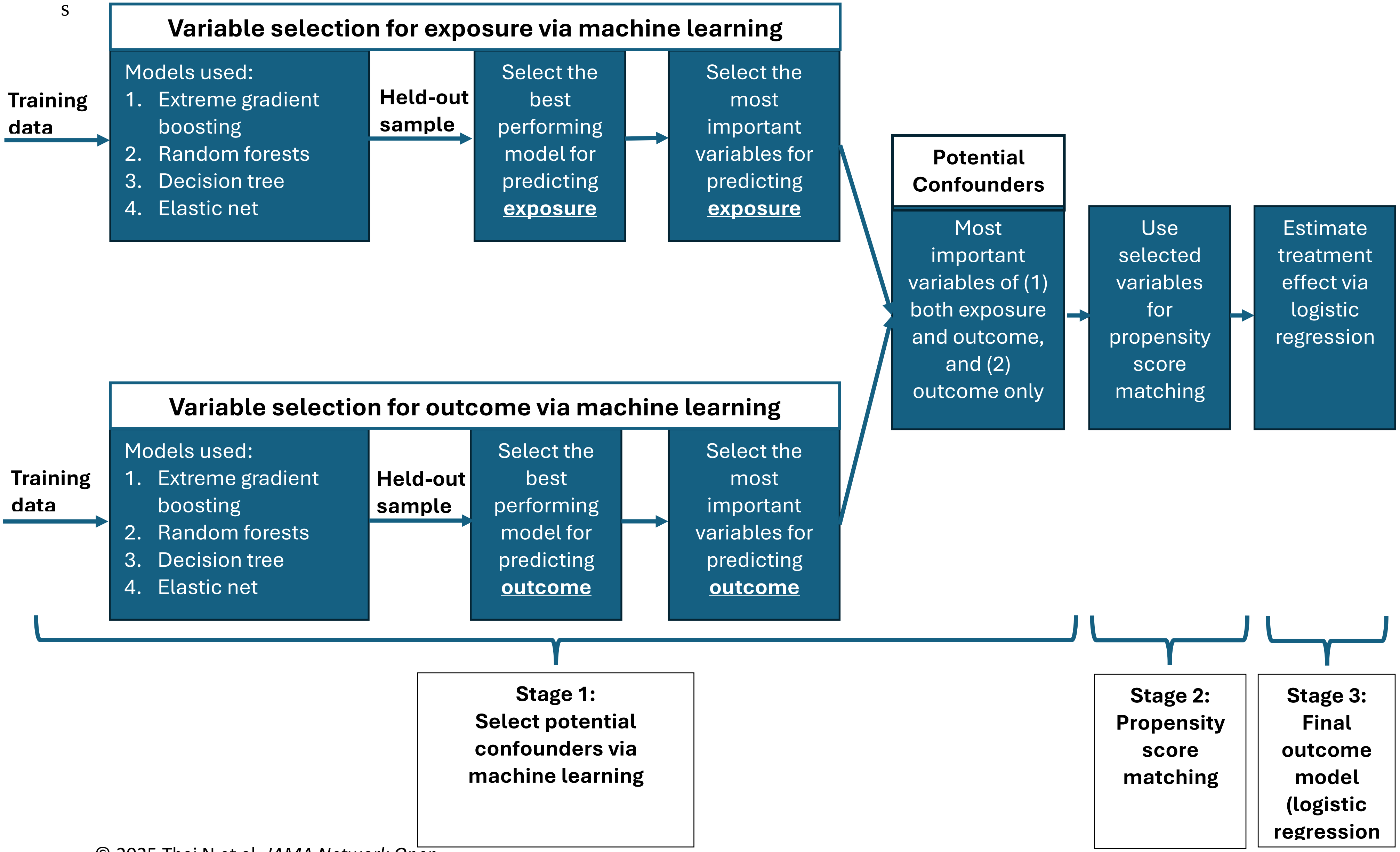

© 2025 Thai N et al. *JAMA Network Open.*

eMethods 2. Using Instrumental Variable Analysis to Estimate the Effect of Integration on Cardiac Rehab Participation

We performed an instrumental variable (IV) analysis to estimate the association between integration and cardiac rehab participation. We identified the level of hospital employment in a county, as measured by a county's proportion of integrated physicians, as the IV for this analysis. There is strong evidence from prior studies that patients are more likely to receive care closer to their place of residence.[3] This suggests that patients who reside in counties with high levels of hospital-employed physicians would be more likely to seek care from hospital-integrated physicians, irrespective of underlying health conditions or other predictors of care patterns. We therefore used our area prevalence measure as an IV in this robustness check against the possibility of unobserved patient selection into treatment from integrated physicians.

We conducted two-stage generalized predictor substitution estimators (TSPS) described in Terza et al. (2008).[4] This method is implemented in the R package OneSampleMR.[5] The R implementation of TSPS performs generalized method of moment (GMM) estimation to ensure appropriate standard errors on its estimates similar to the approach introduced by Clarke et al. (2015).[6] The first stage is a logistic regression model of the exposure regressed upon the instrument and any measured confounders. The second stage is a logistic regression model of the outcome regressed on the predicted values of the exposure from the first stage and any measured confounders.

For this analysis, the study sample consists of 20,403 patients with a known value for the IV (i.e., the proportion of integrated physicians in the county where the patient resides). We included study covariates that were identified as the strongest predictors of cardiac rehab using the machine learning approach described in our main analysis. Our results suggest that having an integrated primary care physician or cardiologist after a cardiac event is associated with a 26 percent increase in the odds of receiving cardiac rehab (odds ratio = 1.26 [95% CI: (1.05, 1.52)]). These results imply that, if anything, the numbers that we present in the main body of the paper are conservative estimates of the effect of integration on receiving cardiac rehab.

© 2025 Thai N et al. *JAMA Network Open.*

eTable 3. Odds Ratios and 95% Confidence Intervals Estimated by the Two-stage Generalized Predictor Substitution Approach for the Primary Study Exposure (i.e., Receiving Care from an Integrated Physician) and other Study Covariates

Variable	OR (95% CI)
**Integrated primary care physician or cardiologist**	1.26 (1.05-1.52)
**CABG at index**	2.98 (2.68-3.31)
**Age**	0.99 (0.99-0.99)
**AMI at index**	0.83 (0.75-0.91)
**Transportation - patient, provider, equipment**	0.44 (0.41-0.48)
**PTCA at index**	1.24 (1.13-1.36)
**Diagnostic cardiac catheterization, coronary arteriography**	1.56 (1.42-1.71)
**Excision of skin lesion**	1.49 (1.39-1.60)
**Region - Northeast**	0.45 (0.41-0.50)
**Region - South**	0.57 (0.53-0.62)
**Region - West**	0.69 (0.60-0.79)
**Physical therapy exercises, manipulation, and other procedures**	1.51 (1.39-1.63)
**Race - Black**	0.61 (0.52-0.70)
**Race - Other[1]**	0.63 (0.52-0.76)
**Race - Unknown**	1.37 (1.04-1.81)
**Female**	0.82 (0.76-0.87)

Abbreviations. AMI, acute myocardial infarction; CABG, coronary artery bypass grafting; CI, confidence interval; OR, odds ratio; PTCA, percutaneous transluminal coronary angioplasty.
[1] Other race category includes: Asian, Hispanic, North American – Native, and others

© 2025 Thai N et al. *JAMA Network Open.*

eTable 4. Full Results of Unadjusted Comparisons between Patients of Hospital-Integrated Primary Care Physicians or Cardiologists and Patients of Independent Primary Care Physicians or Cardiologists

Variable	Patients of Independent PCPs or Cardiologists in Follow-Up, No. (%) (N=19559)	Patients of Integrated PCPs or Cardiologists in Follow-Up, No. (%) (N=9037)	Overall, No. (%) (N=28596)
**All-Cause Inpatient Charges (Pre-Index)**			
High (≥75% Percentile)	4928 (25.2)	2221 (24.6)	7149 (25.0)
Low (<75% Percentile)	2605 (13.3)	1439 (15.9)	4044 (14.1)
None	12026 (61.5)	5377 (59.5)	17403 (60.9)
**CVD Inpatient Charges (Pre-Index)**			
High (≥75% Percentile)	4846 (24.8)	2303 (25.5)	7149 (25.0)
Low (<75% Percentile)	481 (2.5)	259 (2.9)	740 (2.6)
None	14232 (72.8)	6475 (71.6)	20707 (72.4)
**All-Cause Outpatient Charges (Pre-Index)**			
High (≥75% Percentile)	4709 (24.1)	2440 (27.0)	7149 (25.0)
Low (<75% Percentile)	12006 (61.4)	5941 (65.7)	17947 (62.8)
None	2844 (14.5)	656 (7.3)	3500 (12.2)
**CVD Outpatient Charges (Pre-Index)**			
High (≥75% Percentile)	4759 (24.3)	2390 (26.4)	7149 (25.0)
Low (<75% Percentile)	6999 (35.8)	4261 (47.2)	11260 (39.4)
None	7801 (39.9)	2386 (26.4)	10187 (35.6)
**All-Cause Office Charges (Pre-Index)**			
High (≥75% Percentile)	5394 (27.6)	1755 (19.4)	7149 (25.0)
Low (<75% Percentile)	12738 (65.1)	6040 (66.8)	18778 (65.7)
None	1427 (7.3)	1242 (13.7)	2669 (9.3)
**CVD Office Charges (Pre-Index)**			
High (≥75% Percentile)	5598 (28.6)	1551 (17.2)	7149 (25.0)

© 2025 Thai N et al. *JAMA Network Open.*

Low (<75% Percentile)	4132 (21.1)	1362 (15.1)	5494 (19.2)
None	9829 (50.3)	6124 (67.8)	15953 (55.8)
**All-Cause Outpatient Visits (Pre-Index)**			
High (≥75% Percentile)	3930 (20.1)	3646 (40.3)	7576 (26.5)
Low (<75% Percentile)	12785 (65.4)	4735 (52.4)	17520 (61.3)
None	2844 (14.5)	656 (7.3)	3500 (12.2)
**CVD Outpatient Visits (Pre-Index)**			
High (≥75% Percentile)	5172 (26.4)	4146 (45.9)	9318 (32.6)
Low (<75% Percentile)	6586 (33.7)	2505 (27.7)	9091 (31.8)
None	7801 (39.9)	2386 (26.4)	10187 (35.6)
**All-Cause Office Visits (Pre-Index)**			
High (≥75% Percentile)	5702 (29.2)	1774 (19.6)	7476 (26.1)
Low (<75% Percentile)	13246 (67.7)	6492 (71.8)	19738 (69.0)
None	611 (3.1)	771 (8.5)	1382 (4.8)
**CVD Office Visits (Pre-Index)**			
High (≥75% Percentile)	5770 (29.5)	1423 (15.7)	7193 (25.2)
Low (<75% Percentile)	6374 (32.6)	2246 (24.9)	8620 (30.1)
None	7415 (37.9)	5368 (59.4)	12783 (44.7)
**Myocardial Infarction**	2205 (11.3)	1215 (13.4)	3420 (12.0)
**Congestive Heart Failure**	3886 (19.9)	2387 (26.4)	6273 (21.9)
**Peripheral Artery Disease**	3071 (15.7)	1953 (21.6)	5024 (17.6)
**Stroke**	2466 (12.6)	1368 (15.1)	3834 (13.4)
**Dementia**	361 (1.8)	207 (2.3)	568 (2.0)
**Pulmonary Disease**	3272 (16.7)	1950 (21.6)	5222 (18.3)
**Rheumatic Disease**	456 (2.3)	302 (3.3)	758 (2.7)
**Peptic Ulcer Disease**	191 (1.0)	92 (1.0)	283 (1.0)
**Liver Disease (Mild)**	556 (2.8)	324 (3.6)	880 (3.1)
**Diabetes**	3889 (19.9)	1871 (20.7)	5760 (20.1)
**Diabetes with Complications**	2136 (10.9)	1396 (15.4)	3532 (12.4)
**Paralysis**	80 (0.4)	45 (0.5)	125 (0.4)

© 2025 Thai N et al. *JAMA Network Open.*

eTable 4 continued. Full Results of Unadjusted Comparisons between Patients of Hospital-Integrated Primary Care Physicians or Cardiologists and Patients of Independent Primary Care Physicians or Cardiologists

Variable	Patients of Independent PCPs or Cardiologists in Follow-Up, No. (%) (N=19559)	Patients of Integrated PCPs or Cardiologists in Follow-Up, No. (%) (N=9037)	Overall, No. (%) (N=28596)
**Renal Disease**	2934 (15.0)	1789 (19.8)	4723 (16.5)
**Cancer**	1337 (6.8)	816 (9.0)	2153 (7.5)
**Liver Disease (Severe)**	41 (0.2)	33 (0.4)	74 (0.3)
**Metabolic Syndrome**	160 (0.8)	103 (1.1)	263 (0.9)
**HIV**	29 (0.1)	36 (0.4)	65 (0.2)
**History of Cardiac Event during Baseline**	323 (1.7)	168 (1.9)	491 (1.7)

Abbreviations. CVD, cardiovascular disease; HIV, human immunodeficiency virus; PCP, primary care physician.

© 2025 Thai N et al. *JAMA Network Open.*

eTable 5. Model Accuracy Metrics from Machine Learning Algorithms Predicting the Primary Exposure (i.e., Receiving Care from an Integrated Primary Care Physician or Cardiologist)

Model	Recall	Balanced Accuracy	AUC
**Extreme gradient boosting**	0.935	0.806	0.845
**Random forests**	0.921	0.806	0.834
**Decision trees**	0.918	0.799	0.827
**Elastic net**	0.935	0.806	0.845

Abbreviations. AUC, area under the curve.

© 2025 Thai N et al. *JAMA Network Open.*

eTable 6. Model Accuracy Metrics from Machine Learning Algorithms Predicting the Primary Outcome (i.e., Receiving Post-Index Cardiac Rehab Services)

Model	Recall	Balanced Accuracy	AUC
**Extreme gradient boosting**	0.901	0.642	0.700
**Random forests**	0.902	0.628	0.687
**Decision trees**	0.872	0.615	0.654
**Elastic net**	0.909	0.621	0.687

Abbreviations. AUC, area under the curve.

© 2025 Thai N et al. *JAMA Network Open.*

eTable 7. Model Accuracy Metrics from Machine Learning Models Predicting the Secondary Exposure (i.e., the Likelihood of Cardiac Rehab Participation)

Model	Recall	Balanced Accuracy	AUC
**Gradient Boosted Trees**	0.919	0.561	0.627
**Random forests**	0.919	0.559	0.626
**Decision Trees**	0.879	0.564	0.604
**Elastic Net**	0.956	0.547	0.651

Abbreviations. AUC, area under the curve.

© 2025 Thai N et al. *JAMA Network Open.*

eTable 8. Most Important Variables Identified by the Elastic Net Algorithm for Predicting Recurrent Cardiovascular-related Hospitalizations (i.e., the Secondary Outcome)

Ranking	Variable	Overall
**1**	Congestive Heart Failure (pre-index)	33.1%
**2**	CCS Category: Colostomy, temporary or permanent (pre-index)	32.3%
**3**	AMI (index event)	28.4%
**4**	Renal Disease (pre-index)	22.8%
**5**	CCS Category: Lower gastrointestinal X-ray (pre-index)	22.7%
**6**	Diabetes with Complication (pre-index)	19.6%
**7**	CCS Category: Use of Transportation for Emergency/Non-Emergency Care (pre-index)	19.0%
**8**	Low office visit (pre-index)	14.6%
**9**	CCS Category: Radioisotope Pulmonary Scan (pre-index)	14.3%
**10**	CCS Category: Arthroplasty Knee (pre-index)	13.6%
**11**	No CVD inpatient charge (pre-index)	13.4%
**12**	Low CVD inpatient charge (pre-index)	12.0%
**13**	Cardiac Rehab Use (post-index)	11.0%
**14**	CCS Category: Other OR Lower GI Therapeutic Procedures (pre-index)	10.6%
**15**	Diabetes (pre-index)	8.9%
**16**	Peripheral Vascular Disease (pre-index)	8.7%
**17**	CCS Category: Contrast Arteriogram of Femoral and Lower Extremity Arteries (pre-index)	8.2%
**18**	CCS Category: Diagnostic Cardiac Catheterization (pre-index)	8.2%
**19**	CCS Category: Ancillary Services (pre-index)	7.9%
**20**	No Inpatient Hospitalization (pre-index)	7.3%

Abbreviations. AMI, acute myocardial infarction; CCS, Clinical Classification System; CVD, cardiovascular disease; GI, gastrointestinal.

© 2025 Thai N et al. *JAMA Network Open.*

eTable 9. Odds Ratios and 95% Confidence Intervals from Logistic Regression Model for Recurrent Cardiovascular-related Hospitalizations (i.e., the Secondary Outcome)

Variable	OR (95% CI)
**Cardiac Rehab Use (post-index)**	0.86 (0.81-0.91)
**AMI (index event)**	1.45 (1.34-1.56)
**CCS Category: Use of Transportation for Emergency/Non-Emergency Care (pre-index)**	1.32 (1.23-1.41)
**CCS Category: Diagnostic Cardiac Catheterization (pre-index)**	0.80 (0.74-0.87)
**Congestive Heart Failure (pre-index)**	1.47 (1.38-1.58)
**CCS Category: Colostomy, temporary or permanent (pre-index)**	1.51 (0.27- 8.49)
**Renal Disease (pre-index)**	1.32 (1.22-1.43)
**CCS Category: Lower gastrointestinal X-ray (pre-index)**	2.87 (1.17-7.34)
**Diabetes with Complication (pre-index)**	1.24 (1.13-1.36)
**Low office visit (pre-index)**	0.79 (0.74-0.84)
**No office visit (pre-index)**	0.92 (0.80-1.07)
**CCS Category: Radioisotope Pulmonary Scan (pre-index)**	1.55 (1.25-1.93)
**CCS Category: Arthroplasty Knee (pre-index)**	0.83 (0.65-1.06)
**Low CVD inpatient charge (pre-index)**	0.96 (0.80-1.15)
**No CVD inpatient charge (pre-index)**	0.72 (0.65-0.81)
**CCS Category: Other OR Lower GI Therapeutic Procedures (pre-index)**	1.80 (1.05-3.06)
**Diabetes (pre-index)**	1.16 (1.09-1.24)
**Peripheral Vascular Disease (pre-index)**	1.21 (1.13-1.29)
**CCS Category: Contrast Arteriogram of Femoral and Lower Extremity Arteries (pre-index)**	1.54 (0.91-2.64)
**CCS Category: Ancillary Services (pre-index)**	1.15 (1.07-1.23)
**No Inpatient Hospitalization (pre-index)**	0.84 (0.76-0.94)

Abbreviations. AMI, acute myocardial infarction; CCS, Clinical Classification System; CI, confidence interval; CVD, cardiovascular disease; GI, gastrointestinal; OR, odds ratio.

© 2025 Thai N et al. *JAMA Network Open.*

eReferences

1. Ho V, Tapaneeyakul S, Metcalfe L, Vu L, Short, M. Using Medicare data to measure vertical integration of hospitals and physicians. *Health Serv Outcomes Res Methodol.* 2020;20(1):1-12.
2. Neprash HT, Chernew ME, Hicks AL, Gibson T, McWilliams JM. Association of financial integration between physicians and hospitals with commercial health care prices. *JAMA Intern Med*. 2015;175(12), 1932-1939.
3. Hansen BB, Klopfer SO. Optimal full matching and related designs via network flows. *Journal of Computational and Graphical Statistics*. 2006;15(3):609-627.
4. Terza JV, Basu A, Rathouz PJ. Two-stage residual inclusion estimation: addressing endogeneity in health econometric modeling. *J Health Econ*. 2008;27(3):531-543.
5. Palmer T, Spiller W, Sanderson E. OneSampleMR: one sample Mendelian randomization and instrumental variable analyses. https://remlapmot.github.io/OneSampleMR/. Accessed December 5, 2024.
6. Clarke PS, Palmer TM, Windmeijer F. Estimating structural mean models with multiple instrumental variables using the generalised method of moments. *Statistical Science*. 2015;30(1):96-117.

© 2025 Thai N et al. *JAMA Network Open.*
