# Supplementary material for: Hospital-Physician Integration and Cardiac Rehabilitation Following Major Cardiovascular Events
Source: JAMA Netw Open. 2025 Mar 3;8(3):e2462580. doi: 10.1001/jamanetworkopen.2024.62580 (PMC11877175; doi:10.1001/jamanetworkopen.2024.62580)
Supplement: Supplement 2. — Data Sharing Statement [file jamanetwopen-e2462580-s002.pdf]

Data Sharing Statement

Thai. Hospital-Physician Integration and Cardiac Rehabilitation Following Major Cardiovascular Events. *JAMA Netw Open*. Published March 03, 2025.
doi:10.1001/jamanetworkopen.2024.62580

Data
Data available: No

Additional Information
Explanation for why data not available: Data is not publicly available to oblige the data use agreement
